# Clinical findings and management of intraocular foreign bodies (IOFB) in third-world country eye hospital

**DOI:** 10.1186/s12886-025-03903-5

**Published:** 2025-03-18

**Authors:** Made Indra Widyanatha, Henry Santosa Sungkono, Grimaldi Ihsan, Rova Virgana, Erwin Iskandar, Arief Sjamsulaksan Kartasasmita

**Affiliations:** 1 Vitroretinal Department National Eye Center Cicendo Eye Hospital, Bandung, Indonesia; 2 Department of Opthalmology, Universitas Padjadjaran, Bandung, Indonesia

**Keywords:** Intraocular foreign bodies, Trauma, Outdoor activities

## Abstract

**Purpose:**

To describe the demoFigurey and clinical characteristics of intraocular foreign body as open globe injury type at National Eye Center Cicendo Eye Hospital.

**Methods:**

This descriptive-retrospective study is based on medical records of patients admitted to National Eye Center Cicendo Eye Hospital diagnosed with intraocular foreign bodies from January 2019 to June 2023.

**Results:**

A total of twenty-one cases of ocular trauma specifically intraocular foreign bodies were recorded based on medical records. A total of 20 cases (95.2%) were male. The incidence of 15 cases (71.4%) of trauma in outdoor activities with the whole mechanism (100%) of penetrations. Most cases were referred cases (57.1%). 11 cases had surgery under 24 h prior to trauma (52.4%). The entry site of the foreign body was on the cornea (17 cases, 81%). Metallic foreign bodies account for 16 (76.2%); most locations are found on the retina (71.4%). More than three fourths of patients had single IOFB (76.2%). Visual acuity is mostly present between counting fingers- 0.1 Snellen. Patients showed to have had retinal breaks (61.9%).

**Conclusion:**

The majority of IOFB patients were working-age males. The nature of IOFBs is mostly metallic and retained in the posterior segment.

## Introduction

Ocular trauma is an eye emergency and is one of the leading causes of preventable visual impairment morbidity. The object held inside the eyeball in penetration trauma is called an intraocular foreign body [1]. An intraocular foreign body is a type of Open Globe Injury (OGI) in which there is an injury to the entire eyewall [2]. Intraocular foreign bodies occur often in the working age group developed and developing countries. The average age of patients was 36–42 years, and they had an intraocular foreign body incidence rate of 16% of OGI efficacy. The male sex predominates the incidence of intraocular foreign bodies [3]. Trauma mechanisms related to work activities in various countries include mowing grass (64%) and hammering (32.7% and 43%) [4]. Most foreign bodies are metallic, with the posterior eye segment where foreign bodies are most encountered [3–6]. Initial diagnosis and examination of proper care are essential in prognosis and actions against patients with intraocular foreign bodies. Some factors associated with the sharpness of the final vision of patients with intraocular foreign bodies include sharp initial vision, size and location of foreign bodies, size and location of entrance wounds foreign bodies, and concomitant findings such as endophthalmitis [6–8]. To understand the clinical characteristics of patients with intraocular foreign bodies, we conducted a study at the National Eye Center Cicendo Eye Hospital based on data from 2019 to 2023.

## Research methodology

This study used a descriptive observational design. The study data used patient medical records taken retrospectively. This research was conducted at Cicendo Eye Hospital. The study subjects were patients from January 2019 to June 2023. The sample of this study was taken using the total sampling method. The inclusion criteria for this study sample are all patients with intraocular foreign bodies from January 2019 to June 2023 and surgery. Exclusion criteria are patients who refuse surgery and are unable to give consent. The demografic data of the study subjects observed were gender, age, place of trauma, use of eye protection, eye trauma mechanism, laterality of eye trauma, place of first trauma treatment, and time of surgery after the trauma event. Visual acuity showed in LogMar. All data processing uses Microsoft Office Excel 2021 for MacOS. Statistical analysis using univariate analysis for categorical data are proportion and percentage which used in almost variable, except for age that used mean and standard deviation.

## Result

A total of 21 patients were included in the study based on inclusion and exclusion criteria. Table 1 shows demoFigureic data. Out of 21 patients, most are men (95.2%) with an average age of 39.8 ± 13.41 years. A total of 7 patients are in the 30–39 age group. The youngest patient with intraocular foreign bodies is 19, whereas the oldest is 65. Outdoor locations were the location of the most trauma incidents, with 15 (71.4%) while accident place at workplace and indoor are both three patients respectively (14,3%). Patients who did not use eye protection were recorded as many as seven people (33.3%), while the remaining data is unknown. The mechanism of ocular trauma in this study are all due to penetrations. The laterality of right eye trauma was 11 cases (52.4%) compared to 10 in left eye cases (47.6%). The place of first treatment was more carried out in other hospitals, namely as many as 12 people (57.1%) compared to Cicendo Eye Hospital, as many as nine people (42.9%). Surgery after a traumatic event performed in less than 24 h in as many as 11 cases (52.4%). The fastest surgery is done within 2 h after trauma, while the longest is two years after. The average.

**Table 1:** DemoFigureic data of intraocular foreign bodie’s patients

DemoFigureic Data	Total (*n* = 21)	%
**Gender**		
Man	20	95.2
Woman	1	4.8
**Age**		
< 10 year	0	0.0
10–19 year	1	4.8
20–29 year	4	19.0
30–39 year	7	33.3
40–49 year	3	14.3
50–60 year	4	19.0
> 60 year	2	9.5
**Oculi Trauma Accident Place**		
Workplace	3	14.3
Outdoor	15	71.4
Indoor	3	14.3
**Eye Protection Use**		
Yes	0	0
No.	7	33,3
Unknown	14	66,7
**Trauma Oculi Mechanism**		
Penetration	21	100
Rupture	0	0
Perforation	0	0
**Lateralisation**		
Right eye	11	52,4
Left eye	10	47,6
**Place of First Treatment**		
Cicendo Eye Hospital	9	42,9
Other Hospitals	12	57,1
**Time of Surgery after Trauma**		
≤ 24 h	11	52,4
> 24 h	10	47,6

The characteristics of intraocular wounds and foreign bodies are shown in Table 2. The location of foreign body entry was on the cornea (17 cases, 81%) and sclera (4 cases, 19%). The diameter of the foreign body entry wound varies with the smallest size 0.1 mm and the largest 5 mm. The diameter of the most foreign body entry wounds is less than 3 mm (28.6%). Based on its location, intraocular foreign bodies are most prevalent in the retina (15 cases, 71.4%). Most foreign bodies are metallic (16 objects, 76.2%). All foreign objects are metals in the form of iron. Non-metal objects encountered are stones (2 cases) and ceramics (1 case). The highest number of single intraocular foreign bodies extracted was 16 cases (76.2%). The size of objects varies from 0.1 mm to 6 mm, but most are unknown in size.

**Table 2:** Characteristics of wound and intraocular foreign bodies

Patients Data	Total (*n* = 21)	%
**Foreign Bodies on The Outer Layer**		
Cornea	17	81
Sclera	4	19
**Wound Diameter Foreign Bodies Wound**		
< 3 mm	0	0.0
3–5 mm	1	4.8
> 5 mm	4	19.0
Not known	7	33.3
**Foreign Bodies Location**		
Anterior Camera Oculi	1	4.8
Lens	1	4.8
Retina	15	71.4
Vitreous	2	9.5
Not Known	2	9.5
**Material of Foreign Bodies**		
Metal	16	76.2
Non-Metal	5	23.8
**Amount of Foreign Bodies**		
One	16	76.2
More than one	0	0
Not Known	5	23.8
**Size of Foreign Bodies**		
< 1 mm	1	4,8
1–5 mm	0	0
> 5 mm	1	4,8
> 10 mm	0	0
Not Known	19	90,5

**Fig. 1:**
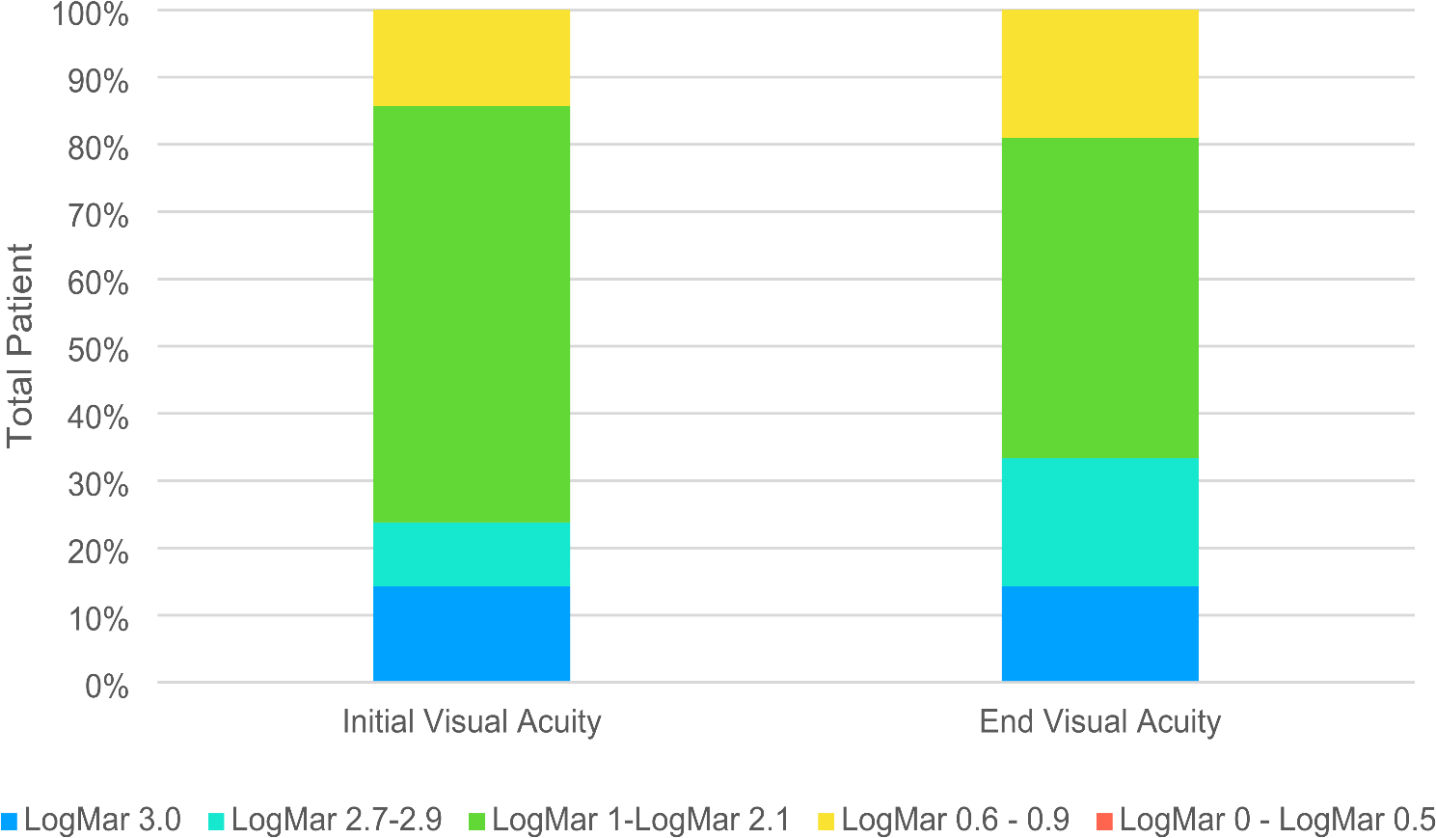
Change of visual acuity in intraocular foreign body patients

Initial and final vision acuity are shown in Fig. 1. All research subjects agreed to surgery. Patients’ initial visual acuity was most prevalent in the LogMAR 1.0–2.1 group with 13 people (61.9%). Final visual acuity was also most prevalent in the same group, LogMAR 1.0–2.1, with ten people (47.6%). A total of 10 people (47.6%) experienced an increase in visual acuity after surgery, while two people (9.5%) did not experience an increase or a decrease.

The most concomitant clinical findings were retinal breaks in 13 cases (61.9%). One patient can have more than 1 accompanying clinical finding. Seven patients were found to have traumatic cataracts and retinal breaks simultaneously. Table 3 presents concomitant clinical findings.

**Table 3:** Concomitant clinical findings of intraocular foreign body patients

Clinical Findings	Sum (*n* = 21)	%
Retinal Break	13	61.9
Traumatic Cataract	12	57.1
Vitreous Hemorrhage	4	19
Retinal Detachment	4	19
Corneal Avulsion	1	4.8
Endophthalmitis	1	4.8
Hyphema	1	4.8

Supporting examinations in ultrasound (USG) were carried out in all subjects. Other supports carried out were Roentgen (5 cases, 23.8%) and CT-Scan (1 case, 4.8%). One person can do more than one supporting examination.

## Discussion

The diagnosis of intraocular foreign body based on the foreign object present inside eyeball that then can make a penetration. The incidence rate of this disease reached up to 16% both in developed and developing countries majority in male age-worker [1]. 

### Epidemiology

In this study, 21 cases of intraocular foreign bodies were found from January 2019 to June 2023. Intraocular foreign body research conducted previously at the National Eye Center of Cicendo Eye Hospitals showed mixed results. Intraocular foreign body cases from July 2014 to December 2017 showed 35 cases, while from January 2017 to January 2022, there were 39 cases. At Dr. Soetomo Surabaya Hospital, in the period 2016–2020, 30 intraocular foreign body patients who followed up six months after trauma were recorded.9–11 The global incidence of intraocular foreign bodies was 46.63 million in 2019 [12]. The incidence of intraocular foreign bodies also increased by 30.29% from 1990 to 2019 [13]. Economic patterns, social factors such as employment, and Government policy relate to the incidence rate of intraocular foreign bodies [12, 13]. 

Male has a greater incidence rate than the female. This study also obtained results from 20 cases (95.2%) of male sex. A consistent finding also showed that previous research by Liu et al. in China reported that of 370 intraocular foreign body cases, 97% of patients were male [4]. Research at Cicendo Eye Hospital in 2014–2017 stated that male sex was 97.1% of the 35 patients studied [9]. Men usually work in industries prone to intraocular foreign body cases, such as construction, welding, carpentry, agriculture, and mining [4, 9, 10, 13]. 

Patients of productive age experience more cases of intraocular foreign bodies than children and the elderly (over 60 years). The average age in this study was 39.8 years, with the age group of 30–39 years being the most significant number of 7 people (33.3%). Research in South Korea by Jung et al. obtained data on the average age of patients, 46.7 ± 15.8 years with an age range of 3 to 74 years [6]. Gea et al. reported the highest age group of intraocular foreign body patients at Cicendo Eye Hospital in the range of 40–49 years (34.3%), followed by 30–39 years (28.6) with an average age of 36.8 years [9]. In Beijing, China, Li et al. stated that of the 1340 patients studied for ten years, the highest age range was in the 30–39 year group, with a total of 335 patients (25%) [14]. 

The location of oculi trauma incidence in our study was highest in outdoor activities at 15 cases (71.4%). Outdoor activities documented covered activity like falling from heights and traffic accidents. Fujikawa et al. reported work-related occupational trauma in 27 eyes (45.8%), followed by falls from a height of 19 eyes (32.2%) [16]. Cases of intraocular foreign bodies occur most often in the workplace. Gea et al. reported that of the 35 patients studied at the National Eye Center of Cicendo Eye Hospitals, 30 patients (85.7%) experienced occupational trauma at work. The most frequent activities associated with intraocular foreign bodies are hammering, metal cutting, gardening, drilling, and sculpting. Intraocular foreign bodies can also cause from explosives, such as fireworks and firearms [4, 17, 18]. 

Penetration is a mechanism of oculi trauma that occurs in all research subjects. Chang et al. reported penetrations to be the most traumatic mechanism in 1050 (88.8%) patients compared to perforation in the 10-year review Southwest China [19]. Gea et al. reported all cases (100%) of intraocular foreign bodies having penetration trauma mechanisms [9]. Rupture and perforation trauma mechanisms have a poorer prognosis [20]. Trauma types can be assessed using the intraocular foreign body prognosis score (OTS) [21]. 

In this study, as many as seven people (33.3%) did not use eye protection. Eye protection was only 6% of cases in 96 patients in the study by Ehlers et al. [22] on metallic intraocular foreign bodies. In another study by Gea et al., 11.4% did not use eye protection, and 88.6% did not know whether to wear it because it was not recorded [9]. Our study’s lateralization of the eyes is different from previous studies. In Hong Kong, of the 21 cases, the incidence of the right eye was 29%, and the left eye was 71%0.17 In the Irish study, 57% of cases occurred in the left eye, while the remaining 43% were in the right eye [23]. Research at Cicendo Eye Hospital for January 2017 – January 2022 showed that the case of the left eye was 51.3% and the right eye was 48.7% [10]. 

The percentage of surgery after trauma of less than 24 h and more than 24 h was about the same in this study. In China, the same was found with a ratio of less than 24 h at 55.9% and more than 24 h at 44.1%0.24 The action’s timing is unrelated to the final vision’s sharpness [25, 26]. Bourke et al. reported that 86.9% of cases (23 eyes) had surgery on the same day they came to the hospital [23]. Zhang et al. reported that the first operation time < 24 h was 415 cases compared to ≥ 24 h, which was 280 cases. The time from trauma to surgery is related to the incidence of endophthalmitis. Intraocular foreign body extraction and eyeball repair within 24 h after trauma in 76.2% of 21 cases resulted in no endophthalmitis events [17]. 

The place of handling patients for the first time is more done in other hospitals based on research by Gea et al., where 57.1% of patients were treated at other hospitals before being referred to Cicendo Eye Hospital [9]. 

In this study, ten people (47.6%) experienced a improvement in their vision after surgery. Another study found that in 70% of cases, visual acuity improved after intraocular foreign-body retrieval [25]. Initial visual acuity in another study by Ma et al. was most in the LP – CF group with 63 out of 73 cases, while final vision acuity was most abundant in the 0.05–0.25 group with 29 cases [32]. Gea et al. reported the most initial vision acuity in the LP-HM group with 18 cases (51.4%) and the final visual acuity in the 0.3-1.0 group (31.4%) [9]. Good initial vision acuity is also associated with good visual output [5, 8, 32, 36]. 

### Characteristics of intraocular foreign bodies and imaging

The location of foreign body entry can be classified into three zones based on the Priamigi et al. that gives a Ocular Trauma Classification Group, namely zone 1 (open wounds limited to the limbus and cornea areas), zone 2 (wounds to the posterior limbus up to 5 mm posterior sclera) and zone 3 (wounds to posterior to 5 mm from the limbus) [27]. The location of foreign body entry in this study is the same as the previous study, where the cornea was the most foreign body entry location with a percentage of 59.6 − 76%. Jung et al. in South Korea reported that the most foreign body penetration sites were in the cornea with 31 eyes (59.6%), followed by the corneosclera (23.1%) and sclera (17.3%) [28]. At the National Eye Center of Cicendo Eye Hospitals, Gea et al. reported the same thing with the location of corneal penetration in 21 cases (60%), followed by sclera (31.4%) and corneoscleral (5.7%). Liu et al. in Hong Kong reported that the cornea is the most entry site for foreign objects with thirteen cases (76%) compared to the sclera (4 cases, 24%). The cornea is also the entry location of foreign bodies at 1145 (67.3%) cases [6, 9, 17, 24, 25, 29, 30]. Foreign bodies entering through the cornea can result in corneal sciatic and have a poor prognosis of vision [16]. 

The diameter of the most foreign body entry wounds in this study was the same as previous research. Gea et al. reported the most wound diameter size < 3 mm with 13 cases (37.1%)0.9 The diameter of the foreign body entry wound has a significant relationship with the risk factor for sharp late vision < 20/200.19 Another study by Watanachai et al. in Thailand also put the wound diameter < 3 Mm at 45.1% of 162 cases [5]. The wound diameter < 3 mm has a significant relationship with visual acuity < 20/400 [5]. Research founds that the larger foreign bodies size reside in the eyeball especially bigger than entry wound diameter correlate to more poor prognosis [31]. 

The location of foreign bodies in this study is the same as in some previous studies, namely on the retina, with a percentage of about 34 − 57.7%. Rozon et al. reported the most foreign body locations on the retina, namely at 31 cases (57%), followed by vitreous at 19 cases (35%). Chang et al. in China reported the retina to be the most common foreign body location in 44.1% of 1176 patients, followed by the vitreous (28.7%). Zhang et al. in China reported that the posterior segment of intraocular foreign bodies was 997 out of 1296 cases.6,19,25,30 The location of foreign bodies on the posterior segment has poorer visual outcomes [8, 31].

The most common foreign body properties found in this study are metals and, according to other studies, with a percentage of 67 − 92.9% [5, 6, 17, 25, 31]. Watanachai et al. mentioned metal objects in as many as 122 cases (75.7%), followed by stone (8.1%), wood (3.1%), and glass (1.9%) [5]. Jung et al. reported that the most foreign objects were metal (76.9%), stone (9.6%), glass (5.8%), pencil tip (3.8%), and others (3.8%) [4]. Of the 21 cases, Liu et al. reported that most metal objects (67%) were followed by glass (5%). At the same time, the rest were unknown [17]. Rozon et al. reported that foreign bodies were mostly metal (89%) followed by glass (6%), while the rest were fireworks, organic, and unknown at 2% each [25]. Ma et al. reported that magnetic foreign objects such as iron flakes (35 cases, 47.95%) and nails (11 cases, 15.7%) were more abundant than non-magnetic objects in large intraocular foreign bodies [32]. The nature of foreign bodies needs to be known because they have different impacts. Objects containing iron (Fe) cause bulb siderosis due to photoreceptors and retinal epithelial pigment damage [33]. Organic foreign bodies such as plants, eyelashes, and insects can increase the risk of endophthalmitis [24]. Foreign objects glass or plastic, for example, has a more negligible risk of damage [24, 34].

The number of intraocular foreign bodies in 1 patient in several previous studies was the same as our findings, with a percentage of 92.7 – 94.1%. Gea et al. at Cicendo Eye Hospital received foreign objects with the most number of 1 encountered, amounting to 97.1% of 35 cases, while 1 case was unknown the number of foreign objects [9]. Chang et al. reported the number of foreign bodies dominating 94.1% of the 1176 cases [19]. Liu et al. Reported 19 (90.4%) cases of the intraocular foreign body. There was one foreign body, while there were two intraocular foreign bodies in the other 2 (9.6%) [17]. A poorly detectable amount of intraocular foreign body will cause severe inflammation, toxicity, and an increased risk of endophthalmitis [35]. The number of intraocular foreign bodies has no relationship to the development of endophthalmitis [15, 24]. 

In our study, the foreign body sizes were not recorded in medical records. Research by Gea et al. states that the size of intraocular foreign objects is the highest in the 1–5 mm range, with a percentage of 17.1% [9]. The size of a small intraocular foreign body is related to external risk factors for sharp final vision [36, 37]. Intraocular foreign body size with the most common size of less than 3 mm (38%) is related to postoperative vision [24]. Intraocular foreign body diameter ≥ 3 mm has poorer visual output [8, 25]. Large intraocular foreign body sizes are associated with complications such as cataracts, hemorrhage, and uvea prolapse [4]. 

In this study, ultrasound was performed on all research subjects. In another study at the National Eye Center of Cicendo Eye Hospitals, ultrasound was performed in 71.4 − 97% of cases [9, 11]. Gea et al. reported the use of ultrasound in 25 cases (71.4%), followed by roentgen 8 cases (22.9%) and CT scan in 2 cases (5.7%). In the study by Desrina et al., ultrasound was performed on 97% of 39 cases, followed by schedule (41%) and CT scan (10%) [10]. B-scan ultrasound is usually used to detect foreign bodies swallowed on the posterior segment. The disadvantage of using an ultrasound B scan is that it depends on the operator’s expertise [38]. CT scans are the most sensitive imaging examination and can show the condition of intraocular foreign objects with a size of 0.5–25 mm. However, CT scans sometimes cannot detect foreign objects with low density and small size [39]. Another thing is that CT-scans are preferable for metallic IOFB with threshold around 0.07 mm^3^. X-rays for intraocular foreign body screening are less valuable than ultrasound and CT scans [40]. 

### Other clinical findings

The most common concomitant clinical findings are retinal break and traumatic cataracts. Traumatic cataracts were also the most common finding (81.3%) in the Chinese study [28]. Retinal break and traumatic cataracts were also the second most common findings (61.5%) after corneal trauma (83.7%) in the South Korean study [6]. Retinal break is one of the factors associated with poor visual output [4]. Damage or loss of retinal photoreceptor cells results in permanent vision damage, particularly involving Macular [16]. In some studies, traumatic cataracts were not associated with end-visual outcomes [4, 8]. 

### Educational measure and prevention

This study includes the evaluation of eye protection use, however majority of patients status for eye protection are unknown. Protective eyewear should be encouraged in risk activities to the incidence of IOFB [42]. As majority of cases happen in outdoor settings that are not related to the workplace, this will be a challenge for educational measures. However, educational measures are needed to prevent the increase of the incident [43]. 

The drawback of this study is the need for more medical record data. Some variables, such as the diameter of the foreign body entrance wound, the location of the foreign body, the number of foreign objects, and the size of the foreign body, should be recorded in the patient’s medical record. Data that show the relationship between variables with each other is a shortcoming in this study. This study suggests that with a larger population and more complete data be conducted.

## Conclusion

This study included 21 patients that documented from January 2019 to June 2023 at National Eye Center, Cicendo Eye Hospital. The epidemiology of this case presented most in male (95.2%) in working age group 30–39 years old (33,3%). Trauma occurs from outdoor activities from more than three-quarter patients. All the trauma mechanism are penetrations with mostly metallic foreign bodies (76.2%) in the posterior segment of retina (71.4%). Common complications presented with the intraocular foreign bodies were retinal break. Study showed 21 patients from January 2019 to June 2023 at PMN Cicendo Eye Hospital, with the most being male (20 people, 95.2%) and in the age group of 30–39 years (7 people, 33.3%). Most trauma cases occurred outdoors (15 cases, 71.4%), with the entire trauma mechanism (21 cases, 100%) being penetration. Foreign bodies are mostly metallic (16 objects, 76.2%), with the most locations in the posterior segment being the retina (71.4%). The most common complication in the study was a retinal break (61.9%).

This study is the first study conducted in four years documented all intraocular foreign bodies case in third-world country national eye hospital and can be a reference for establishing the diagnosis and management of intraocular foreign body patients in Indonesia.

## Data Availability

The data that support the findings of this study are available from the corresponding author, H.S, upon reasonable request.
